# Coenzyme Q10 suppresses apoptosis of mouse pancreatic β-cell line MIN6

**DOI:** 10.1186/s13098-018-0351-4

**Published:** 2018-06-14

**Authors:** Keisuke Sumi, Tsuyoshi Okura, Youhei Fujioka, Masahiko Kato, Takeshi Imamura, Shin-ichi Taniguchi, Kazuhiro Yamamoto

**Affiliations:** 1 Division of Endocrinology and Metabolism, Molecular Medicine and Therapeutics, Faculty of Medicine, Tottori University, 36-1 Nishi-chou, Yonago, Tottori 683-8504 Japan; 2 Division of Cardiovascular Medicine, Endocrinology and Metabolism, Department of Molecular Medicine and Therapeutics, Faculty of Medicine, Tottori University, Yonago, Tottori 683-8504 Japan; 3 Division of Molecular Pharmacology, Faculty of Medicine, Tottori University, Yonago, Tottori 683-8504 Japan; 4 Department of Regional Medicine, Faculty of Medicine, Tottori University, Yonago, Tottori 683-8504 Japan

**Keywords:** MIN6, Coenzyme Q10, Staurosporine, Apoptosis, Mitochondrial diabetes

## Abstract

**Background:**

In mitochondrial diabetes, apoptosis of β-cells caused by mitochondrial stress plays an important role in impaired insulin secretion. Several studies have reported that coenzyme Q10 (CoQ10) has therapeutic effects on mitochondrial diabetes, but no reports have examined the fundamental effectiveness or mechanism of CoQ10 in mitochondrial diabetes. We previously reported in a Japanese article that CoQ10 has protective effects on pancreatic β-cells against mitochondrial stress using mouse pancreatic β-cell line MIN6 and staurosporine (STS). Here, we report that CoQ10 protects MIN6 cells against apoptosis caused by STS and describe the more detailed apoptotic cascade.

**Methods:**

Apoptosis of MIN6 cells was induced by 0.5 µM STS treatment for specific periods with or without 30 μM CoQ10. The apoptosis cascade in MIN6 cells was then investigated using WST-8 assays, annexin-V staining, western blotting, and DNA degradation analysis.

**Results:**

Sixteen hours of 0.5 μM STS treatment led to 47% cell viability, but pretreatment with 30 μM CoQ10 resulted in significantly higher viability of 76% (P < 0.01). CoQ10 also prevented translocation of phosphatidylserine from the inner leaflet to the outer leaflet of the cell membrane. CoQ10 prevented cytochrome c release from mitochondria and activation of caspase-3.

**Conclusion:**

We concluded that CoQ10 protects pancreatic β-cells through anti-apoptotic effects against STS treatment.

## Background

Hyperglycemia occurs because of impaired insulin action in diabetes as a result of insulin resistance and impaired insulin secretion. In insulin resistance, although insulin is secreted, its effect decreases. Conversely, in impaired insulin secretion, insulin secretion itself decreases. Mitochondria are important for insulin secretion of pancreatic β-cells. Impaired insulin secretion occurs in mitochondrial diabetes, a hereditary disorder of mitochondrial functions [1]. Mitochondrial diabetes accounts for 1% of diabetes in Japan. Although insulin secretion depends on the function and quantity of pancreatic β-cells, these cells have a low proliferation ability, and their quantitative and qualitative decreases are strongly affected by apoptosis [2]. Apoptosis of β-cells is caused by oxidative stress, lipotoxity, and cytokines. Furthermore, it has been reported that pancreas β-cells decrease as a result of apoptosis in BHE/cdb rats, a model of mitochondrial diabetes [3]. These results suggest that mitochondrial dysfunction and apoptosis have an important role in impaired insulin secretion. Other than insulin injection, treatments have not been established for mitochondrial diabetes, although several studies have shown that coenzyme Q10 (CoQ10) has therapeutic effects on mitochondrial diabetes [4]. However, to the best of our knowledge, no reports have examined the fundamental effectiveness or mechanism of CoQ10 in mitochondrial diabetes. Reactive oxygen species produced by mitochondria cause mitochondrial disorders [5]. A report has demonstrated the effectiveness of CoQ10 to treat a central symptom of mitochondrial disease [6]. The antioxidant and ATP supplying effects of CoQ10 may protect central nerves [7]. Therefore, the mechanism of CoQ10 treatment in mitochondrial diabetes might be protective effects from apoptosis of pancreatic β-cells. We previously reported in a Japanese article that CoQ10 has protective effects on pancreatic β-cells against mitochondrial stress using mouse pancreatic β-cell line MIN6 and staurosporine (STS), a strong inducer of apoptosis and mitochondrial stress by producing reactive oxygen species [8, 9]. Here, we report that CoQ10 protects MIN6 cells against apoptosis caused by STS and describe the apoptotic cascade.

## Methods

### Materials

Mouse insulinoma cell line MIN6 was kind gift from Dr. Makoto Shigeto (Division of Diabetes and Endocrinology Department of Medicine Kawasaki Medical School), Kohei Kaku (Division of Diabetes and Endocrinology Department of Internal Medicine Kawasaki Medical School), and Jun-ichi Miyazaki (Division of Stem Cell Regulation Research, Osaka University Graduate School of Medicine). Fetal bovine serum (FBS) was obtained from Thermo Fisher Scientific, Inc. (Kanagawa, Japan). Phosphate buffered saline (PBS), Dulbecco’s modified Eagle’s medium (DMEM), penicillin, and streptomycin were obtained from Sigma-Aldrich, Inc. (St. Louis, MO). CoQ10 (oxidized form) was obtained from Yokohama Oils & Fats Industry (Kanagawa, Japan). Z-VAD-FMK (carbobenzoxy-valyl-alanyl-aspartyl-[*O*-methyl]-fluoromethylketone) was obtained from Peptide Institute, Inc. (Osaka, Japan). STS and polyacrylamide gels were obtained from Wako Pure Chemical Industries (Osaka, Japan). A protease inhibitor cocktail was obtained from BioVision, Inc. (Milpitas, CA). Tris/Glycine/SDS electrophoresis buffer was obtained from Bio-Rad Laboratories, Inc. (Hercules, CA). Western Blotting Detection Reagent was obtained from GE Healthcare (Tokyo, Japan). Polyvinylidene fluoride (PVDF) microporous membranes were obtained from Merck Millipore (Billerica, MA). Cell lysis buffer, an anti-β-actin antibody, anti-rabbit IgG, and annexin V assay kit were obtained from MBLCo., Ltd. (Aichi, Japan). An anti-caspase-3 antibody was obtained from Cell Signaling Technology (Tokyo, Japan). A cytochrome c releasing apoptosis assay kit was obtained from Funakoshi Co., Ltd. (Tokyo, Japan). An apoptotic DNA ladder kit was obtained from Roche Diagnostics (Tokyo, Japan). Cell Counting Kit-8 was obtained from Dojindo Laboratories (Kumamoto, Japan).

### Cell culture

MIN6 cells were cultured in 60 ml flasks, 96-well plates, or on glass coverslips in DMEM (high-glucose; 4.5 g/l) with 15% FBS, 75 μg/ml penicillin, and 50 μg/ml streptomycin at 37 °C in a humidified atmosphere with 5% CO_2_. At 80% confluence, the cells were divided into four groups: CoQ10, Z-VAD, STS, and control. The CoQ10 group was treated with 30 μM CoQ10 for 4 h before induction of apoptosis. Z-VAD-FMK is a pan-caspase inhibitor and anti-apoptotic agent. The Z-VAD group was treated with 30 μM Z-VAD-FMK for 1 h before induction of apoptosis. STS and control groups were not pre-treated. Except for the control group, each group was treated with 0.5 μM STS for the specified periods to induce apoptosis.

### WST-8 assay

Cell viability was compared among the four groups after apoptotic stimulation using Cell Counting Kit-8 that determines cell viability through reduction of s water-soluble tetrazolium salt, WST-8. WST-8 is reduced by dehydrogenases in cells to produce a yellow formazan dye. The amount of formazan dye generated by the activities of dehydrogenases in cells is directly proportional to the number of living cells. Except for the control group, MIN6 cells were treated in 96-well plates (100 μl medium per well) with 0.5 μM STS for 16 h. Then, 10 μl Cell Counting Kit-8 solution was added to each well, followed by incubation for 2 h. The number of living cells was determined by absorbance at 450 nm using fluorescence microplate reader. Several experiments were performed, and average values were calculated.

### Annexin-V staining

At the early stage of apoptosis, cells lose their phospholipid membrane asymmetry and expose phosphatidylserine (PS) at the cell surface. This process can be monitored using annexin-V, a Ca^2+^-dependent, phospholipid-binding protein with high affinity for PS, which is useful for identifying apoptotic cells with exposed PS. For annexin-V staining, MIN6 cells were cultured on glass coverslips. After 6 h of treatment with STS, cells were washed with PBS and then stained with green fluorescent protein-labeled annexin-V for 5 min while protected from light, according to the manufacturer’s protocol. The cells were then washed with PBS and treated with methanol for 10 min. The methanol was removed, and propidium iodide (PI) was added, a red fluorescent nuclear counterstain. Samples were observed under a fluorescence microscope. Methanol-treated samples (all cells) were stained with PI (red), whereas early apoptotic cells were stained with annexin-V (green) and PI. We counted respectively 140, 113, 208 and 235 cells in Control, STS, CoQ10 and Z-VAD group. Thus, we were able to calculate the ratio of early apoptotic cells.

### SDS-polyacrylamide gel electrophoresis (PAGE) and western blot analysis

To detect activation of caspase-3 and cytochrome c release from mitochondria after treatment with STS, cells were washed and then lysed in cell lysis buffer containing a protease inhibitor cocktail. The cell lysates (10 µg protein per sample) were then separated by 12.5% SDS-PAGE and electrotransferred onto a PVDF transfer membrane. Membranes were blocked with Tris-buffered saline containing with 0.05% Tween 20 and 5% nonfat dried milk and then incubated with primary antibodies for 1 h at room temperature. Primary antibodies were against caspase-3 (1:1000 dilution), cytochrome c (1:200 dilution), and β-actin (1:1000 dilution). The membrane was then incubated with secondary antibodies for appropriate times (30–60 min). Specific proteins were visualized by the enhanced chemiluminescence method using western blotting detection reagent.

### DNA degradation analysis

After 14 h of treatment with STS, isolation of DNA from cells of each group was performed according to a standard procedure using a commercially available apoptotic DNA ladder kit. DNA fragmentation analysis was performed using a 1.5% agarose gel.

### Statistical analysis

Data are expressed as the mean ± standard deviation. Comparisons of individual treatments in WST-8 assays were conducted using the Games-Howell post hoc test following one-way analysis of variance. Comparisons of individual treatments in annexin-V analysis were conducted using the Chi square test. P < 0.05 was considered as statistically significant.

## Results

### Comparing of cell viability

**Fig. 1:**
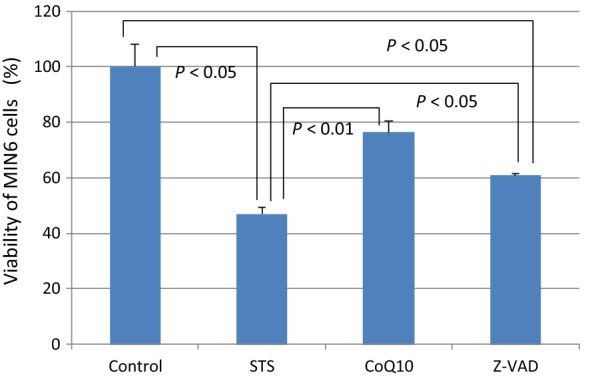
Comparison of cell viability among the four groups after apoptotic stimulation using WST-8 assays. Control, MIN6 cells cultured in regular medium; STS, cells exposed to STS (0.5 µM for 16 h); CoQ10, cells pretreated with CoQ10 (30 µM for 4 h) and then exposed to STS (0.5 µM for 16 h); Z-VAD, cells pretreated with Z-VAD-FMK (30 µM for 1 h) and then exposed to STS (0.5 µM for 16 h). Comparisons of individual groups were conducted using the Games-Howell post hoc test following one-way analysis of variance

We first examined cell viability after STS treatment using WST-8 assays (Fig. 1). After 16 h of STS treatment, the STS group had 47% cell viability, but the CoQ10 group had significantly higher viability of 76% (P < 0.01 vs. STS group). The Z-VAD group had 60% cell viability (P < 0.01 vs. STS group). These results indicate that CoQ10 has a protective effect on MIN-6 cells against STS treatment, and CoQ10 has the same or a stronger protective effect than Z-VAD-FMK.

### DNA fragmentation

**Fig. 2:**
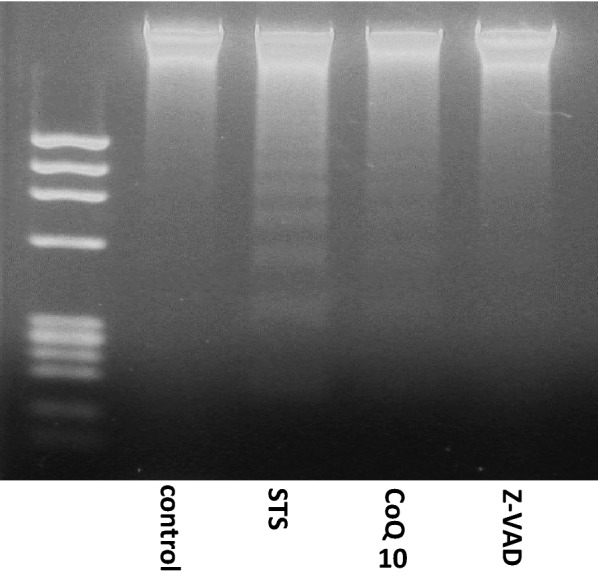
CoQ10 protects MIN6 cells from apoptosis induced by STS treatment. Control, MIN6 cells cultured in regular medium; STS, cells exposed to STS (0.5 µM for 14 h); CoQ10, cells pretreated with CoQ10 (30 µM for 4 h) and then exposed to STS (0.5 µM for 14 h); Z-VAD, cells pretreated with Z-VAD-FMK (30 µM for 1 h) and then exposed to STS (0.5 µM for 14 h). DNA fragmentation was observed in the STS group, which was inhibited by CoQ10 and Z-VAD

Next, we examined DNA fragmentation to determine whether cell death was caused by apoptosis (Fig. 2). Electrophoresis of genomic DNA from MIN6 cells treated with STS for 14 h revealed the characteristic laddering pattern, indicating that MIN6 cell death was caused by apoptosis. However, DNA fragmentation was inhibited by CoQ10 and Z-VAD, suggesting that CoQ10 had a protective effect against apoptosis.

### Annexin-V analysis

**Fig. 3:**
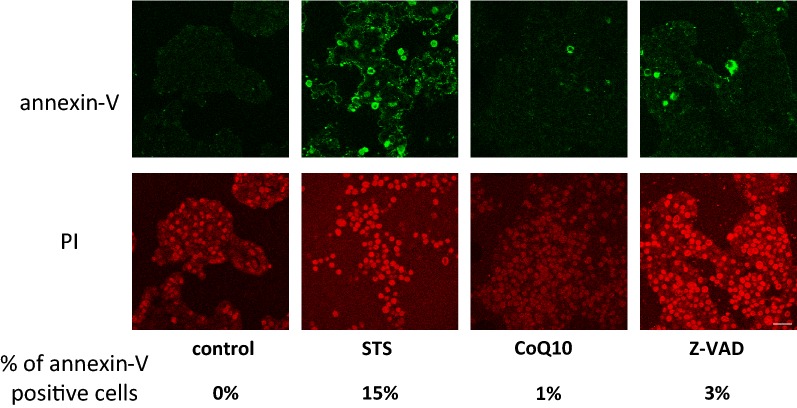
CoQ10 prevents early events of apoptosis induced by STS treatment. Control, MIN6 cells cultured in regular medium; STS, cells exposed to STS (0.5 µM for 6 h); CoQ10, cells pretreated with CoQ10 (30 µM for 4 h) and then exposed to STS (0.5 µM for 6 h); Z-VAD, cells pretreated with Z-VAD-FMK (30 µM for 1 h) and then exposed to STS (0.5 µM for 6 h). Annexin-V-positive cells indicate exposure of PS at the cell surface, the early stage of apoptosis (green). Cells treated with methanol were PI positive (scale bar is 100 µm)

Next, we investigated the detailed apoptosis cascade. During apoptosis, characteristic biochemical changes are observed. Translocation of PS from the inner leaflet to the outer leaflet of the cell membrane is one of the characteristic biochemical changes in the early stage of apoptosis. We investigated exposure of PS using annexin-V staining together with PI staining. After 6 h of STS treatment, 15% of cells in the STS group were positive for annexin-V. Conversely, only 1% of cells in the CoQ10 group and 3% of cells in the Z-VAD group were positive for annexin-V (Fig. 3).

### Release of cytochrome c

**Fig. 4:**
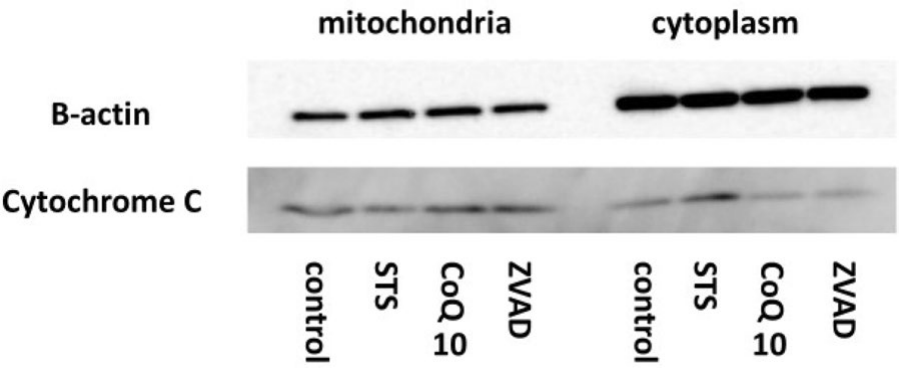
CoQ10 suppresses cytochrome c release from mitochondria. Control, MIN6 cells cultured in regular medium; STS, cells exposed to STS (0.5 µM for 14 h); CoQ10, cells pretreated with CoQ10 (30 µM for 4 h) and then exposed to STS (0.5 µM for 14 h); Z-VAD, cells pretreated with Z-VAD-FMK (30 µM for 1 h) and then exposed to STS (0.5 µM for 14 h). In the STS group, cytochrome c release was high. This change was suppressed by CoQ10

Cytochrome c release from mitochondria is a central event in apoptotic signaling. Cytochrome c usually exists between mitochondrial inner and outer membranes, but it is released to the cytoplasm by apoptotic stimulation. Then, cytochrome c together with Apaf-1 activate caspase-9, leading to the apoptotic caspase cascade. We investigated the release of cytochrome c from mitochondria by western blotting using a commercially available kit. After 14 h of STS treatment, cytochrome c release was increased in the STS group. However, cytochrome c release in the other groups was lower than that in the STS group (Fig. 4). These results indicated that cytochrome c was released into the cytoplasm from mitochondria by STS treatment.

### Caspase-3

**Fig. 5:**
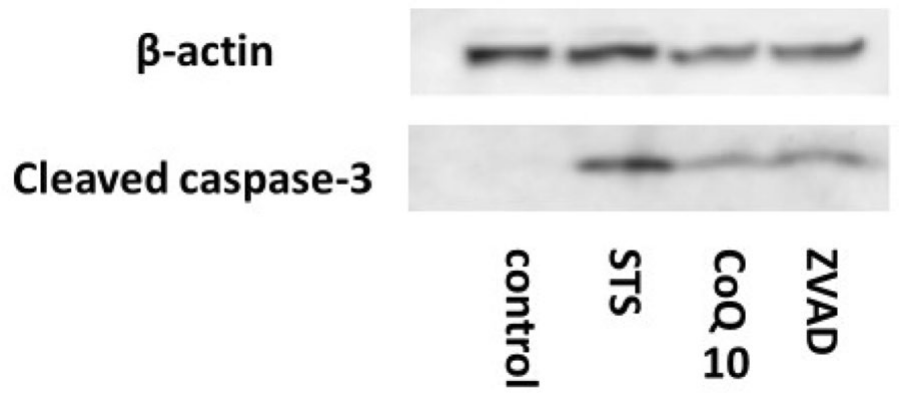
CoQ10 suppresses activation of caspase-3 induced by STS treatment. Control, MIN6 cells cultured in regular medium; STS, cells exposed to STS (0.5 µM for 12 h); CoQ10, cells pretreated with CoQ10 (30 µM for 4 h) and then exposed to STS (0.5 µM for 12 h); Z-VAD, cells pretreated with Z-VAD-FMK (30 µM for 1 h) and then exposed to STS (0.5 µM for 12 h). Cleaved caspase-3 is the active form of caspase-3. STS induced activation of caspase-3, but CoQ10 suppressed this activation

We also investigated activation of caspase-3 by western blotting. After 12 h of STS treatment, cleaved caspase-3, which is the activated form of caspase-3, was strongly detected in the STS group. Conversely, activation of caspase-3 was inhibited in CoQ10 and Z-VAD groups (Fig. 5).

## Discussion

In the pathophysiology of diabetes including mitochondrial diabetes, apoptosis of β-cells plays important roles in impaired insulin secretion. Although the detailed fundamental mechanism of CoQ10 in mitochondrial diabetes is unknown, therapeutic effects of CoQ10 on mitochondrial diabetes have been reported in several studies [4]. We hypothesized that CoQ10 has protective effects against apoptosis in pancreatic β-cells. In this study, we induced apoptosis of MIN6 cells using STS, a mitochondrial stress agent [9]. In WST-8 assays, 0.5 µM STS treatment for 16 h induced the death of about half of the cells. Although cell death might be caused by both apoptosis and necrosis, we showed translocation of PS from the inner leaflet to the outer leaflet of the MIN6 cell membrane, release of cytochrome c from mitochondria, activation of caspase-3, and DNA fragmentation of MIN6 cells. Therefore, MIN6 cell death induced by STS treatment was caused by apoptosis in this study. Based on these results, β-cells were protected by CoQ10 from apoptosis caused by STS treatment. In addition, this protective effect was equivalent to that of Z-VAD-FMK, an anti-apoptotic agent, suggesting that CoQ10 may be an effective anti-apoptotic agent. The fundamental effectiveness or mechanism of CoQ10 in mitochondrial diabetes have not been reported. However, the clinical effectiveness of CoQ10 against a central symptom of mitochondrial disease has been reported [6]. The mechanism is an antioxidant action and stimulates ATP supply from mitochondria [7]. In our study, CoQ10 had an anti-apoptotic action in β-cells. We believe that the protective mechanism of CoQ10 is a direct effect on mitochondria through ATP supply, but elucidation of the detailed mechanism is needed in the future. In mitochondrial diabetes, a hereditary disorder of mitochondrial functions causes impaired insulin secretion [1]. Conversely, in type-2 diabetes, mitochondrial dysfunction caused by various kinds of stress, such as oxidative stress, plays important roles in the pathological progression [10]. Hodgson et al. [11] reported that CoQ10 treatment improves blood pressure and glycemic control in type-2 diabetes patients. This finding suggests that CoQ10 may have protective effects on pancreatic β-cells against mitochondrial stress in the clinic. In Japan, CoQ10 as a ubiquinone is applicable to insurance for heart failure, but not diabetes. However, CoQ10 is already generally used as a supplement. Therefore, if evidence that CoQ10 has effective effects on mitochondrial or type-2 diabetes accumulates, CoQ10 may be adopted clinically.

There are limitations in our study. First, STS treatment is non-physiological stimulation. Further studies are needed to clarify whether CoQ10 has protective effects against apoptosis induced by physiological stimulation. Second, we only investigated MIN6 cells. Experiments using an animal model are necessary to examine the clinical effectiveness of CoQ10. However, we revealed a part of the underlying mechanism of CoQ10 in protection of pancreatic β-cells from apoptosis caused by mitochondrial stress. Elucidation of the more detailed mechanism may facilitate clinical application of CoQ10.

## Conclusion

CoQ10 protects pancreatic β-cells through anti-apoptotic effects against STS treatment.

## Abbreviations

MIN6: mouse insulinoma 6
CoQ10: coenzyme Q10
STS: staurosporine
ATP: adenosine triphosphate
FBS: fetal bovine serum
PBS: phosphate buffered saline
DMEM: Dulbecco’s modified Eagle’s medium
Z-VAD-FMK: carbobenzoxy-valyl-alanyl-aspartyl-(*O*-methyl)-fluoromethylketone
PVDF: polyvinylidene fluoride
DNA: deoxyribonucleic acid
WST-8: water soluble tetrazolium salts 8
PS: phosphatidylserine
PI: propidium iodide
SDS: sodium dodecyl sulfate
PAGE: polyacrylamide gel electrophoresis
